# Supplementary material for: Role and mechanism of NCAPD3 in promoting malignant behaviors in gastric cancer
Source: Front Pharmacol. 2024 Apr 22;15:1341039. doi: 10.3389/fphar.2024.1341039 (PMC11070777; doi:10.3389/fphar.2024.1341039)
Supplement: Supplementary file 11 [file DataSheet2.ZIP › GSEA/Oncogenic signature/my_analysis.Gsea.1599461962066/PIGF_UP.V1_UP.html]

Details for gene set PIGF\_UP.V1\_UP[GSEA]

|  || Dataset | filtered\_dataset.sample\_info.cls#WT\_versus\_NCAPD3\_MUT |
| Phenotype | sample\_info.cls#WT\_versus\_NCAPD3\_MUT |
| Upregulated in class | WT |
| GeneSet | PIGF\_UP.V1\_UP |
| Enrichment Score (ES) | 0.22723916 |
| Normalized Enrichment Score (NES) | 1.0468148 |
| Nominal p-value | 0.4051282 |
| FDR q-value | 0.64570206 |
| FWER p-Value | 1.0 |
Table: GSEA Results Summary

  

Fig 1: Enrichment plot: PIGF\_UP.V1\_UP      
 Profile of the Running ES Score & Positions of GeneSet Members on the Rank Ordered List

  

| SYMBOL | TITLE | RANK IN GENE LIST | RANK METRIC SCORE | RUNNING ES | CORE ENRICHMENT || 1 | 51351 | ZNF117 | 14 | 1.052 | 0.0597 | Yes |
| 2 | 23272 | FAM208A | 20 | 1.032 | 0.1247 | Yes |
| 3 | 55687 | TRMU | 128 | 0.774 | 0.0987 | Yes |
| 4 | 10795 | ZNF268 | 168 | 0.710 | 0.1177 | Yes |
| 5 | 5189 | PEX1 | 200 | 0.676 | 0.1402 | Yes |
| 6 | 145389 | SLC38A6 | 202 | 0.673 | 0.1842 | Yes |
| 7 | 26278 | SACS | 205 | 0.669 | 0.2272 | Yes |
| 8 | 9674 | KIAA0040 | 315 | 0.586 | 0.1874 | No |
| 9 | 34 | ACADM | 530 | 0.457 | 0.0631 | No |
| 10 | 9653 | HS2ST1 | 591 | 0.429 | 0.0482 | No |
| 11 | 5567 | PRKACB | 595 | 0.426 | 0.0744 | No |
| 12 | 23212 | RRS1 | 681 | 0.387 | 0.0387 | No |
| 13 | 178 | AGL | 711 | 0.368 | 0.0422 | No |
| 14 | 8803 | SUCLA2 | 767 | 0.340 | 0.0251 | No |
| 15 | 4285 | MIPEP | 815 | 0.307 | 0.0115 | No |
| 16 | 6303 | SAT1 | 850 | -0.233 | 0.0024 | No |
| 17 | 3673 | ITGA2 | 858 | -0.264 | 0.0149 | No |
| 18 | 5723 | PSPH | 859 | -0.267 | 0.0327 | No |
| 19 | 571 | BACH1 | 884 | -0.300 | 0.0353 | No |
| 20 | 4052 | LTBP1 | 928 | -0.353 | 0.0277 | No |
| 21 | 7503 | XIST | 967 | -0.385 | 0.0258 | No |
| 22 | 57213 | SPRYD7 | 1103 | -0.480 | -0.0399 | No |
| 23 | 9236 | CCPG1 | 1128 | -0.500 | -0.0240 | No |
| 24 | 4189 | DNAJB9 | 1223 | -0.598 | -0.0522 | No |
| 25 | 5997 | RGS2 | 1336 | -0.770 | -0.0820 | No |
| 26 | 4919 | ROR1 | 1385 | -0.954 | -0.0533 | No |
| 27 | 6782 | HSPA13 | 1394 | -1.064 | 0.0116 | No |
Table: GSEA details [plain text format]

  

Fig 2: PIGF\_UP.V1\_UP      
 Blue-Pink O' Gram in the Space of the Analyzed GeneSet

  

Fig 3: PIGF\_UP.V1\_UP: Random ES distribution      
 Gene set null distribution of ES for **PIGF\_UP.V1\_UP**
